# Size distribution of ring polymers

**DOI:** 10.1038/srep27661

**Published:** 2016-06-15

**Authors:** Shlomi Medalion, Erez Aghion, Hagai Meirovitch, Eli Barkai, David A. Kessler

**Affiliations:** 1 Department of Physics, Bar-Ilan University, Ramat-Gan, 52900, Israel; 2 Institute of Nanotechnology and Advanced Materials, Bar-Ilan University, Ramat-Gan, 52900, Israel

## Abstract

We present an exact solution for the distribution of sample averaged monomer to monomer distance of ring polymers. For non-interacting and local-interaction models these distributions correspond to the distribution of the area under the reflected Bessel bridge and the Bessel excursion respectively, and are shown to be identical in dimension *d* ≥ 2, albeit with pronounced finite size effects at the critical dimension, *d* = 2. A symmetry of the problem reveals that dimension *d* and 4 − *d* are equivalent, thus the celebrated Airy distribution describing the areal distribution of the *d* = 1 Brownian excursion describes also a polymer in three dimensions. For a self-avoiding polymer in dimension *d* we find numerically that the fluctuations of the scaled averaged distance are nearly identical in dimension *d* = 2, 3 and are well described to a first approximation by the non-interacting excursion model in dimension 5.

## Introduction

In nature, chemically identical copies of a random polymer come in different shapes and sizes due to their vast number of conformational states. Traditionally, ensemble averaged observables, like the averaged radius of gyration, are used to quantify the size of a polymer, concealing the ever-present fluctuations^1^,^2^. In recent years a new set of single polymer experiments have succeeded in determining the structure fluctuations^3^,^4^,^5^,^6^ of a polymer, one snapshot at a time. This has led to the investigation of fluctuations of single polymers. For example based on a random walk picture, a single realization of a polymer is prolate, while from symmetry, the ensemble average shape is spherical^7^. The conformational fluctuations of open polymers can be analyzed using the theory of random walks (RW)^1^,^8^,^9^. While much is known about fluctuations in linear polymer chains, much less is known about ring polymers (see, however, Refs. 6,10 which studies the size distribution of small unknotted vs. knotted rings of different complexities). It is therefore interesting to study the fluctuation properties of these objects, to test how the additional constraint of closure modifies the size statistics.

Ring polymers^2^ are commonly found in many biological systems, e.g., bacterial and mitochondrial genomes, as well as DNA plasmids^10^,^11^,^12^ used in many molecular biology experiments^13^,^14^,^15^. Recently, ring polymers were also studied in the context of a model for chromosome territories in the nucleus of eukaryotic cells^16^ and of a model for cell-division in bacteria^17^. In particular, the fluctuations of the polymer size are of physical and biological importance, since size controls packing properties and reaction rates, among other things.

In the current paper we study the distribution of sample-averaged monomer-to-monomer distance of ring polymers, both for ideal, noninteracting polymers as well as for polymers with excluded volume. In particular, we exploit recent mathematical developments on *d* dimensional constrained Brownian motion (defined below)^18^ to find an exact expression for the distribution of the sample-averaged monomer to monomer distance, both for ideal ring polymers and those with an additional excluded volume interaction applied at a single point. This observable yields insight into the size fluctuations of polymer rings. The resulting distributions are then compared numerically to those measured in simulations for both the above two cases and, in addition, to simulations with full excluded volume constraints. We find that the excluded volume effects change, as expected, the overall size of the ring polymer, but also the distribution shape. Surprisingly, we find that interactions act as an effective shift of dimensionality, reducing the fluctuations considerably.

As we have noted, constrained random walks lie at the heart of our analysis. For rings, the primary constraint is that the path returns to the origin after *N* steps. Statistics of such constrained Brownian paths in one dimension have been the subject of much mathematical research^19^,^20^,^21^,^22^. These constrained paths have been given various names, depending on the additional constraints imposed. The basic case is that of a Brownian bridge, where the return to the origin is the sole constraint. Paths which are also forbidden from reaching the origin in between the endpoints are called Brownian excursions. Majumdar and Comtet used Brownian excursions to determine statistical properties of the fluctuating Edwards-Wilkinson interface in an interval^23^,^24^. The focus of most previous work has been on constrained one dimensional Brownian paths (note that the problems of non-intersecting Brownian excursions^25^ or vicious random walkers^26^ in higher dimensions are exceptions). The case of the fluctuations of ring polymers requires the extension of the theory of Brownian excursions and bridges to other dimensions. We address the influence of different kinds of interactions on the polymer structure, both analytically (for a single point interaction) and numerically (for a polymer with excluded volume interactions). These models yield rich physical behaviors and open new questions.

### Polymer Models

**Figure 1:**
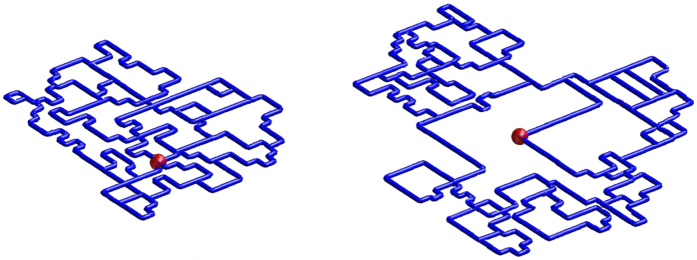
Artistic representation of ideal (left) and LIR (right) model configurations. The LIR model is forbidden from having self-intersections at the “origin”, and the configuration is drawn larger than its ideal partner to indicate its typically larger size.

We consider three lattice models of ring polymers with *N* bonds, each of length *b*, in *d* dimensions. The simplest polymer model is an “ideal ring” - a closed chain without excluded volume^8^, where different monomers can occupy the same lattice site. An ideal ring (IR) corresponds exactly to an unbiased random walk in *d* dimensions which starts and ends at the origin, i.e. a *d*-dimensional bridge. In the “local interaction ring (LIR) polymer” model, the first and last monomers are tied to the origin and no other monomer is allowed to occupy this lattice site. The differences between these two models is exemplified in Fig. 1. The LIR case is equivalent to that of *d*-dimensional excursions. The third model is a ring polymer with excluded volume interactions, also called a self-avoiding walk (SAW). Further details on the models and simulation methods are provided in Supplementary A. We first consider the ideal and local interaction ring models, for which we can provide analytic solutions.

### Bessel process

In the analogy between statistics of an ideal polymer and a RW, the position of the *i*th monomer, **r**_*i*_, corresponds to the position **r**_*i*_ of the random walker after *i* time steps. In the Brownian (large number of steps, *N*) limit, *N* is proportional to the total observation time. The Bessel process^27^,^28^ describes the dynamics of the distance *r* = |**r**| from the origin of a Brownian particle in *d* dimensions. This process is described by the following Langevin equation:


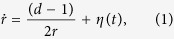


where *η*(*t*) is Gaussian white noise satisfying 〈*η*〉 = 0 and 〈*η*(*t*)*η*(*t*′)〉 = *δ*(*t* − *t*′). One may map the polymer models to the Bessel process using 〈**r**(*t*)^2^〉 = *dt* = *Nb*^2^ = 〈**R**^2^〉, where **R**^2^ is the mean squared end-to-end distance of an ideal linear polymer chain without constraints. In what follows we choose *b*^2^ = *d* and *t* = *N*.

### Bessel Excursions and Reflected Bridges

The process *r*(*t*) with the additional constraint of starting and ending at the origin, is called a reflected (since *r* ≥ 0) Bessel bridge. This process corresponds to an ideal (non-interacting) ring chain. Bessel excursions are paths still described by Eq. (1), with the additional constraint that any path that reaches the origin (not counting the endpoints *t* = 0 and *t* = *N*) is excluded. The Bessel excursion corresponds to what we have called the “local interaction” ring, where a multiple occupation of the origin is not allowed. The mapping of the polymer models to the Bessel process, allows us to extract statistical properties of the former with new tools developed in the stochastic community^18^,^24^,^29^.

### The Observable A

For linear polymers, two observables are traditionally studied. The first is the radius of gyration, *R*_*g*_. This is the observable most easily accessed in experimental studies of polymer ensembles. However, for studies of the fluctuations, it is preferable to consider the end-to-end distance, which is trivially Gausian in the ideal case. Similarly, for a ring polymer, it is difficult to obtain analytically the distribution for *R*_*g*_. While the end-to-end distance obviously has no meaning for a ring polymer, there is a new observable whose distribution, as we will see, is analytically tractable. Let **r**_*i*_ be the position of the *i*th, (*i* = 0, …, *N*) monomer in space and we place the origin at the position of the zeroth monomer, *r*_0_. In the LIR model, this monomer is also the excluding one. This new observable, *A*, is defined by


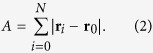


In the RW language, *A* is the area under a random (non-negative) process, and hence is a random variable itself. Clearly 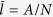 is the sample averaged distance of the monomers from the origin. Specifically, let the area under the random Bessel curve *r*(*t*) be denoted by 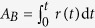 (the subscript *B* is for Bessel). More generally, the mapping of the processes implies that, in the limit of large *N*, the distribution of *A*_*B*_/〈*A*_*B*_〉 is identical to the distribution of *A*/〈*A*〉 (or 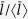), with the corresponding constraints. We note that unlike some other observables, such as the distance to the (*N*/2)^*th*^ mer^15^, which have been suggested as a measure of polymer size, *A* can only be small if the entire polymer ring is collapsed, similar to what happens in the case of the radius of gyration.

## Results

### Numerical results

**Figure 2:**
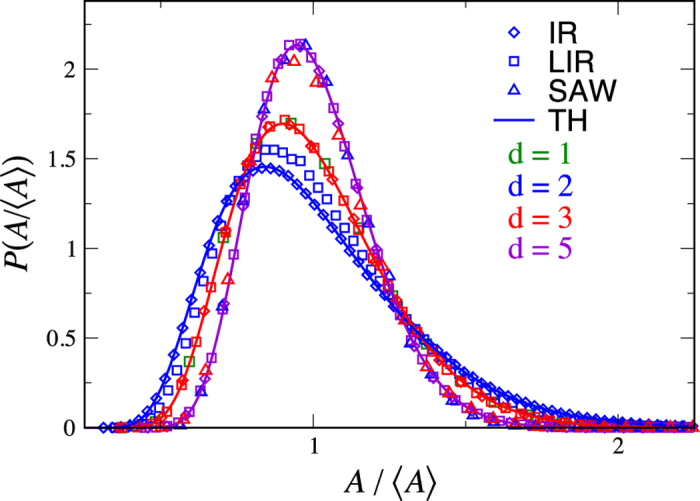
Theoretical *P*^+^(*A*/〈*A*〉)Eq. (10) in dimensions *d* = 3, 5 nicely matche simulations of the ideal ring (IR) and the local interaction (LIR) models. In *d* = 2, however, agreement with theory for the local interaction model is poorer due to critical slowing down (the simulations did not converge for *N* = 10^6^, see text). The theoretical curves (solid lines) for the two models are identical for *d* ≥ 2. The *d* = 1 LIR theory is identical to the *d* = 3 theory, which the simulation confirms. The SAW simulations in *d* = 2, 3 are practically indistinguishable from each other and from the theoretical curve corresponding to an ideal ring in dimension *d* = 5. The shape of the symbol indicates the model simulated (square = LIR, diamond = IR, triangle = SAW). The lines correspond to theory. The color indicates the dimension (green = 1d, blue = 2d, red = 3d, violet = 5d).

To build some intuition for the problem we first present numerical results, which will be later compared to theory. In Fig. 2 we plot the probability density function (PDF) of the scaled random variable *A*/〈*A*〉. For reference, one can compare it to the PDF of the radius of gyration of open chains^30^. For dimensions *d* = 2, …, 5, there is a clear trend of narrowing of the PDF for increasing *d*. This trend is explained by examining Eq. (1): As *d* increases, the noise term becomes negligible compared with the force term, resulting in smaller fluctuations and narrower tails. Against this expected trend are the results in *d* = 1 for the local interaction model. Figure 2 exhibits a second surprising feature. As our analytical calculations will confirm, the local interaction model in dimensions one and three are seen to have the same distribution even though *d* = 1 has a vanishing deterministic force term in Eq. (1) while for *d* = 3 the force is clearly not zero. In addition, we observe that for *d* ≥ 3 the shape of the distributions of locally interacting and ideal ring chains coincide, indicating that weak interactions are negligible (when *N* → ∞). As we shall see, this is also observed in the theory. Indeed the theory discussed below suggests that these two distributions are already identical for *d* = 2. However, this is a critical dimension, with extremely slow convergence, due to logarithmic corrections^31^ to scaling for the LIR case, so we don’t see this behavior in the simulations^32^. This is treated in more detail below. As for the SAW polymer, we see that the fluctuations are considerably reduced compared to the other models. This is due to the fact that small conformations are rejected from the sample, since they overlap and thus fluctuations are reduced. A striking observation is that the two and three dimensional SAW results are identical, both being equal to the simulations of the *d* = 5 IR and LIR models. We now present a theoretical analysis in order to address these observations.

### Functionals of Constrained Bessel Processes

Our goal is to find the PDF *P*(*A*_*B*_, *t*) of the functional 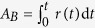 of the Bessel process, constrained to start and end at the origin. The advantage of the observable *A* is that it is a linear functional of the path, unlike other observables such as the radius of gyration. We show that the difference between the local interaction model (the Bessel excursion) and the ideal polymer (the reflected Bessel bridge) enters through the boundary condition in the Feynman-Kac type of equation describing these functionals. The choice of boundary condition turns out to be non-trivial and controls the solution. Other aspects of the solution follow the steps in Ref. 18, and so our description here will be brief.

It is useful to find first the Laplace transform of *P*(*A*_*B*_, *t*), i.e., 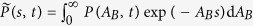 to solve the Feynman-Kac equations, and invert 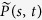 back to *P*(*A*_*B*_, *t*). Let *G*_*t*_(*r, A*_*B*_|*r*_0_) be the joint PDF of the random pair (*r, A*_*B*_) with initial condition *G*_0_(*r, A*_*B*_|*r*_0_) = *δ*(*A*_*B*_)*δ*(*r* − *r*_0_) and 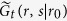 its Laplace pair. The Feynman-Kac equation deals with fluctuations of functionals of Brownian motion (*d* = 1 in Eq. (1)). Here, we have to deal with the effective restoring force introduced by higher dimensionality, and this requires a generalized Feynman-Kac equation. The modified Feynman-Kac equation reads^33^:


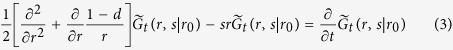


with 
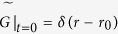
 and *r*_0_ is the initial radius which serves to regularize the calculation and is eventually taken to zero, since excursions are defined to start at the origin. For *d* = 1, the force in Eq. 1 is absent, and so the second term on the left hand side vanishes, and we get the celebrated Feynman-Kac equation corresponding to Brownian functionals^34^. The third, linear, term 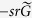 stems from the choice of our observable, namely our functional *A*_*B*_ is linear in *r*^33^. Since we are describing a ring polymer, the Bessel process must start and end at the origin, and so, following Ref. 23, we need to calculate


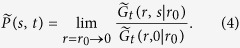


The denominator gives the proper normalization condition.

The first step in the calculation is to perform a similarity transformation:


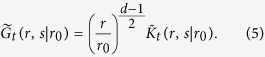


Using Eq. (3), 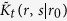 is the imaginary time propagator of a Schrödinger operator:


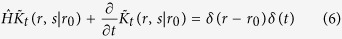


with the effective Hamiltonian:


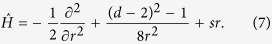


The effective Hamiltonian reveals a subtle symmetry, namely two systems in dimensions *d*_1_ and *d*_2_ satisfying *d*_1_ + *d*_2_ = 4 behave identically. This explains the above-mentioned identity of the *d* = 1 and *d* = 3 PDFs observed in the simulations, a symmetry noted previously in the mathematical literature^35^. Note that this symmetry is not affected by the choice of functional (or observable) since the latter only modifies the last term in 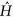.

### Boundary Conditions for Ideal and local Interaction Models

The solution of Eq. (6)


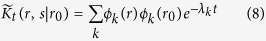


is constructed^18^ from the eigenfunctions *ϕ*_*k*_ of 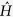 where *λ*_*k*_ is the *k*th eigenvalue and the normalization condition is 
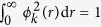
. The subtle point in the analysis is the assignment of the appropriate boundary condition corresponding to the underlying polymer models we consider. The eigenfunctions at small *r* exhibit for  *d* ≠ 2 one of two behaviors:


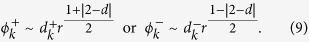


From the normalization condition, the *ϕ*^−^ solution cannot be valid for *d* ≥ 4 and *d* ≤ 0. For the critical dimension *d* = 2 the two solutions are: 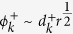 or 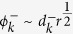 ln *r*. We now solve the problem for the two possible leading boundary behaviors and then show how to choose the relevant one for the physical models under investigation.

### The distribution of *A*/〈*A*〉

Following the Feynman-Kac formalism described above and performing the inverse Laplace transform along the lines of ref. 18, (and for convenience dropping from now on the subscript *B* from *A*, since *A*_*B*_ = *A* in the large *N* limit) we find two solutions for the PDF of the scaled variable *χ* ≡ *A*/〈*A*〉


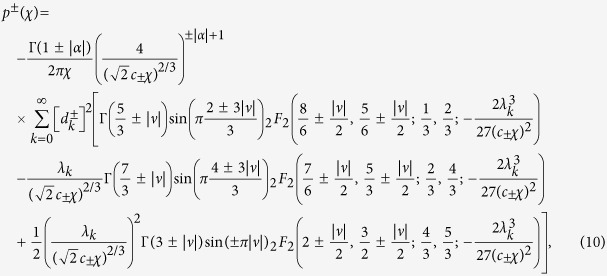


where


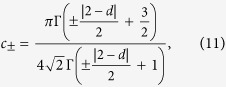


and we have substituted *t* = *N* as explained above. This solution is independent of *N* and valid in the limit of *N* → ∞. Here, |*α*| = |*d* − 2|/2, |*ν*| = 2|*α*|/3, and _2_*F*_2_(⋅) refers to a generalized hypergeometric function. Supplementary B provides a list of *λ*_*k*_ and *d*_*k*_ values for *d* = 1,…,4. For *d* = 1 the solution agrees with the known results^23^,^24^,^36^,^37^, where the + solution is the celebrated Airy distribution^23^,^24^. The average of *A* is


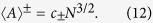


The scaling 〈*A*〉 ∝ *N*^3/2^ is expected since *r* scales with the square root of *N* as for Brownian motion, so the integral over the random processes *r* scales like *N*^3/2^.

**Figure 3:**
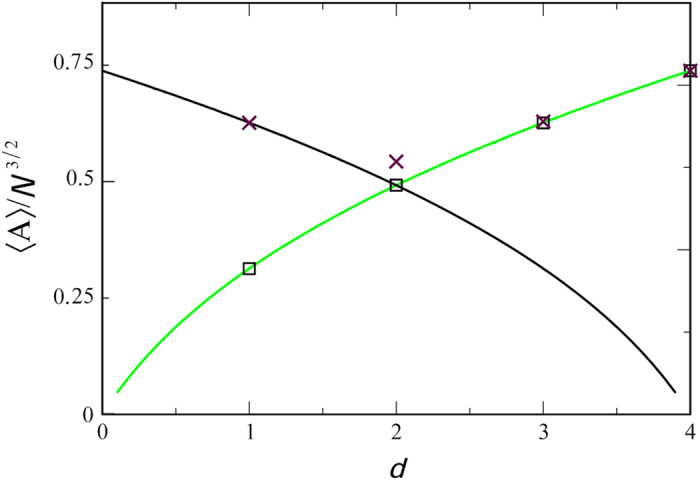
The theory for the normalized average area, 〈*A*〉/*N*^3/2^, under the Bessel excursion (solid black line) and reflected Bessel bridge (solid green line), provided by the + and − branches of Eq. (12), respectively. *N* is the number of steps in the random walk. The simulation results for the IR (black squares) and LIR (brown crosses) models for *d* = 1, 2, 3, 4 matches our expectations, namely a good fit to theory for all but the LIR case at *d* = 2, which do not converge even for *N* = 10^6^, as this is the critical dimension for this case.

The question now is how to choose the solution corresponding to our various polymer models. Clearly, for *d* = 2, 〈*A*〉^+^ = 〈*A*〉^−^, indicating that this is a critical dimension. Further 〈*A*〉^+^ in 1 and 3 dimensions are identical and so is 〈*A*〉^−^ as the result of the symmetry around *d* = 2 in Eq. (7). In Fig. 3, we plot the scaled average area 〈*A*〉/*N*^3/2^, found from the simulation of IR and LIR polymers in different dimensions d = 1, 2, 3, 4 versus the theory provided in Eqs (11, 12). Notice the simulation results for the IR case fit nicely the + branch of Eq. (12), whereas the LIR case agrees with + solution for *d* > 2 and the − solution for *d* < 2. The reason for this agreement is explained immediately below. In *d* = 2, the simulation results for the LIR polymer did not converge even for *N* = 10^6^, which is a direct result of the extremely slow convergence at the critical dimension. We discuss that as well in more detail below.

We investigate the physical interpretation of the two possible boundary conditions. A mathematical classification of boundary conditions was provided in Refs. 28,38 and here we find the physical situations where these conditions apply. We examine the behavior of the probability current associated with the *k*^*th*^ mode through the continuity equation


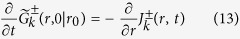


for the *kth* mode of the total probability, 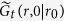 to reach *r* at time t, starting from *r*_0_. We obtain, using Eq. (3),


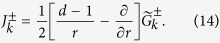


Near the boundary 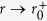, the function 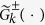 is given by Eqs (5) and (8),


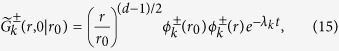


so that, for small *r, r*_0_, (see also ref. 28)


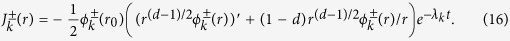


The behavior of this has to analyzed separately for the ± modes, for each value of *d*. This analysis is summarized in Table 1. We see that in dimension two and higher, the current at the origin is either zero or positive. A positive current at the boundary means that probability is flowing into the system, which is an unphysical situation in our system. Hence we conclude that in dimension two and higher, the − solution is not relevant. This implies that the statistics of excursions and reflected bridges (and equivalently, ideal and locally interacting ring polymers) are identical for *d* ≥ 2 and correspond to the + solution. In 1 dimension, the negative current means an absorbing boundary, hence the the + solution describes the excursion (and the LIR polymer). Zero current means a reflecting boundary, so the − solution describes the reflected bridge (IR).

**Figure 4:**
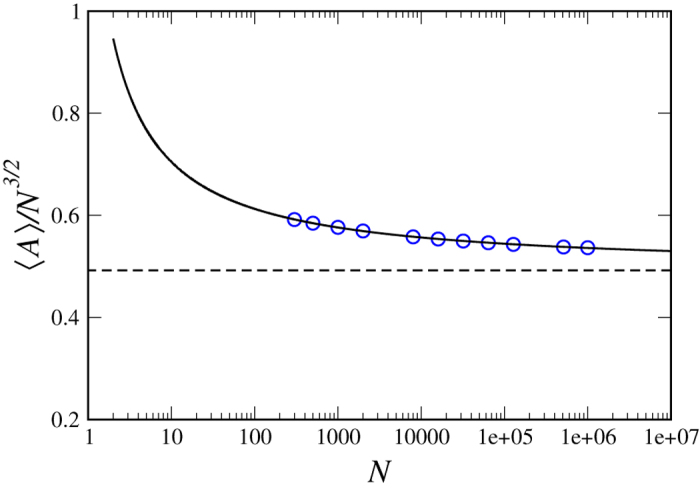
〈*A*〉/*N*^3/2^ as a function of *N* for the critical dimension *d* = 2: local interaction ring (LIR) model simulations (blue circles) and the fitted curve of the function: 〈A〉/*N*^3/2^ ≃ 0.497 + 0.544/log(*N*). The black dashed line is the theoretical value of *c*^+^ = 0.4922 for the *N* → ∞ limit.

In Fig. 2 we compare the results of the simulations of the ideal and locally interacting polymer models with our theoretical results for *P*^+^(*A*/〈*A*〉), as given in Eq. (10). As noted above, for *d* ≥ 3, we see that even for finite size chains the local interaction is not important, and that the theory and simulations perfectly match, while for *d* = 2 there are strong finite size effects in the locally interacting case. This critical slowing down in *d* = 2 is analyzed in Supplementary A and we find logarithmic convergence to our asymptotic result, as can be seen in Fig. 4. The fact that *d* = 2 is critical is surely related to Polya’s problem, since in *d* = 3 the random walk is not recurrent, indicating that local interaction is asymptotically of no importance.

### Self-avoiding polymers

Extensive simulations of ring SAWs were performed on cubic lattices. The global expansion of a polymer of length *N* scales like 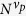, where *ν*_*p*_ is a dimension dependent critical exponent. Since *A* constitutes a measure of the overall size of a polymer, its behavior for large *N* should follow 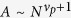. For the ideal and locally interacting chains *ν*_*p*_ = 1/2, and *A* ~ *N*^3/2^. For SAWs the exact value of the exponent depends on *d*, i.e., *ν*_*p*_ = *ν*_*p*_(*d*). *ν*_*p*_ = 1, 0.75, and 0.5 are known exactly for *d* =1, 2, and 4^39^, respectively. For *d* = 3, based on renormalization group considerations and Monte Carlo simulations, 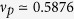
40. These predictions were extensively tested numerically for the observable of interest *A* with a critical dimension of *d* = 4, characterized by a very slow convergence of the locally interacting model (see Supplementary B). While field theoretic calculations includes knotted as well as unknotted rings^41^, the scaling exponent is the same for the subensembles of knotted and unknotted rings (though the corrections to scaling differ^42^).

While the scaling behavior of the SAW model is different from that of the other two models (as reflected in *ν*_*p*_), as we have noted, the scaled PDFs, *P*(*A*/〈*A*〉) are nevertheless similar. A striking observation is that the SAWs in *d* = 2 and 3 coincide to the precision of our measurements with the comparably narrow PDF of the *d* = 5 non-interacting model, (see Fig. 2). That these distributions are narrower than the non-self-avoiding case can be qualitatively explained by the fact that small rings are suppressed in the SAW case due to the need to avoid overlaps.

### Generality of the method

The mapping of ring polymer models to the reflected Bessel bridge and excursion is very promising since it implies that not only the distribution of the size, i.e., *A*, can be analytically computed, but also the statistics of other properties of ring polymers. An example would be the maximal distance from one of the monomers to any other monomer, since that would relate to extreme value statistics of a correlated process. As mentioned in the introduction, the shape of a polymer is prolate^7^. To investigate fluctuations in shape one may consider, for a 2 − *d* polymer, the distribution of the order parameter, 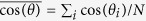, where cos(*θ*_*i*_) = *x*_*i*_/*r*_*i*_, measured from some point on the polymer, which for a circular object is zero. In the language of Brownian motion this quantity is a functional of the random path. Hence we may write a modified Feynman-Kac equation for such an observable and map the problem to a Schrödinger-like Eq. (7). We leave the detailed analysis to future work. Clearly the approach presented here is valuable for calculation of different types of single polymer fluctuations, which as mentioned in the introduction are nowadays accessible in the laboratory.

## Summary

We have quantified analytically and numerically the distribution of the fluctuations of the size of ring polymers, with the hope that these can now be experimentally tested. The famed Airy distribution describes both the one dimensional ideal ring polymer, as well as the three dimensional one, due to the symmetry we have found in the underlying Hamiltonian. The case of *d* = 2 is critical in the sense that interaction at the origin becomes negligible, though for finite size chains it is still important. Boundary conditions of the Feynman-Kac equation were related to physical models, which allowed us to select the solutions relevant for physical models. The SAW polymer exhibits interesting behavior; the distribution of *A*/〈*A*〉 is identical (up to numerical precision) in dimension 2 and 3 and corresponds to the non-interacting models in dimension 5. In general, interactions are seen to reduce the fluctuations.

## Additional Information

**How to cite this article**: Medalion, S. *et al*. Size distribution of ring polymers. *Sci. Rep.* **6**, 27661; doi: 10.1038/srep27661 (2016).

## Supplementary Material

Supplementary Information

## Figures and Tables

**Table 1:** Behavior of the eigenfunctions 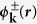 and probability currents 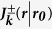 in the proximity of the origin (*r, r*_0_ ≪ 1) for the +/− solutions in different dimensions.

***d***	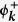	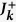	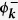	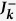
*d* = 1	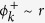	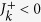	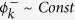	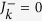
*d* = 2	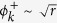	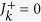	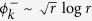	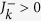
*d* = 3	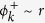	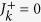	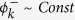	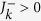

A current *J*^−^ > 0 at the origin is unphysical hence the critical dimension where local interactions are unimportant is 2.
