# Supplementary material for: Size distribution of ring polymers
Source: Sci Rep. 2016 Jun 15;6:27661. doi: 10.1038/srep27661 (PMC4908590; doi:10.1038/srep27661)
Supplement: Supplementary Information [file srep27661-s1.pdf]

Size distribution of ring polymers

Shlomi Medalion, Erez Aghion, Hagai Meirovitch, Eli Barkai, David A. Kessler

Supplementary A: Ring Polymer Simulations in d dimensions

In the main text, we defined three different models of ring polymers. In the ideal case, the only interaction is the constraint on nearest-neighbor distances between particles along the chain as well as the closure constraint. In the second, the Locally Interacting Ring (LIR) model, there is an addition restriction that no point on the chain can be co-located with the origin (and so also terminus) of the chain. In the Self-Avoiding Walk (SAW) model, no two points on the chain may be co-located. In this section, we discuss implementation details of the three models.

A.1 Ideal and Locally Interacting Models

In our simulations for the ideal and weakly-interacting ring polymers the chain is built of $N+1$ consecutive bonds on a lattice. In the ith step, the bond displacement is $\Delta r_{i,j} = \pm 1$ in each of the d directions, $j = 1,\ldots,d$, hence the bond length is $b = \sqrt{\sum_{j=1}^{d}(\Delta r_j)^2} = \sqrt{d}$. For example, for $d = 3$, starting from the origin ($i = 0$) with first step of $\Delta\mathbf{r} = (+1,-1,+1)$ (yielding $b = \sqrt{3}$) we reach the lattice site $\mathbf{r}_1 = (+1,-1,+1)$. For a second step of $\Delta\mathbf{r} = (-1,-1,-1)$ we end up at lattice site $\mathbf{r}_2 = (0,-2,0)$ for the $i = 2$ monomer.

In order to maintain the closure condition of the chain, we choose an array of length of N with $N/2$ components of $(+1)$ and $N/2$ of (-1) in each of the directions (d such arrays), and then shuffle them for each direction separately. For each i, the components of our d dimensional step are the ith values of these arrays. The sum of all of the displacements in each direction is then naturally zero so that the last monomer is always positioned at the origin. For the ideal chain model we built 10^6 such conformations while for the weakly interacting we threw away all the conformations that crossed the origin prior to the final monomer. For each of the conformations we calculated $A = \sum_{i=0}^{N}|r_i - r_0|$, where $r_0 = 0$, and plotted the distribution of this parameter.

According to Eq. (11) in the paper, for $N \to \infty$ we have $\langle A\rangle = c_{\pm}N^{3/2}$. By this we can check the convergence of the simulations to the theory as a function of N. At the critical dimension, $d = 2$ this convergence becomes very slow. In Fig. **??** we plot $\langle A\rangle/N^{3/2}$ for different N values (in a logarithmic scale) in $d = 2$, where $\langle A\rangle = c_+ = 0.4922$ is the theoretical value for $N \to \infty$. One can observe the very slow logarithmic convergence to this value.

A.2 Self-Avoiding Ring Polymers

Monte Carlo (MC) simulations have been applied to self-avoiding ring polymer models[1] on ($d = 2$) square, ($d = 3$) simple cubic, and $d = 4$ hyper-cubic lattices. The polymer consists of N monomers (and N bonds), where the first monomer is attached to the origin of the coordinate system on the lattice, and the Nth monomer is the nearest-neighbor to the origin. At step j of the MC process, monomer k ($1 \le k \le N-1$) is selected at random (i.e., with probability $1/(N-1)$) and the segment of m monomers following k (i.e., $k+1, k+2, , k+m$) become subject to change in the MC process; the rest of the chain (i.e., monomers 1 to k and $k+m+1$ to N) is held fixed (notice that if k is at the end of the chain, $N-m+1 < k \le N-1$, m decreases correspondingly from $m-1$ to 1). Thus, this current segment is temporarily removed and a scanning procedure is used to calculate all the possible segment configurations of m monomers satisfying the excluded volume interaction and the loop closure condition (i.e., the segment of m monomers should start at k and its last monomer, $k+m$ is a nearest neighbor to monomer $k+m+1$; notice that the initial segment configuration is generated as well). The segment configuration for step j is chosen at random out of the set of $\mathscr{L}$ configurations generated by the scanning procedure and the MC process continues.

This process starts from a given ring configuration, whose transient influence is eliminated by a long initial simulation, which leads to typical equilibrium chain configurations. Then, every certain constant amount of MC steps the current ring configuration is stored in a file to create a final sample of n rings from which the averages and fluctuations of the physical properties of interest are calculated. The segment sizes used are $m = 10$ for the $d = 2$ square lattice, $m = 8$ for the $d = 3$ simple cubic lattice, and $m = 4$ and $m = 6$ for $d = 4$. For each lattice several chain lengths, N are studied. Note that while the initial ring structure in each simulation is unknotted, the generated structured during the simulation might become knotted. However, the effect on the results is expected to be insignificant since previous simulations have shown that, if $\overline{R} = BN^{\nu_p}(1+x+\ldots)$, B and ν_p are the same for knotted and unknotted rings, while the difference appears only in the correction to scaling x.[2]

We calculated the ensemble averages of R_g^2,

$$R_g^2 = \frac{1}{N}\sum_{i=0}^{N-1}(\mathbf{r}_i - \mathbf{r}_{c.m.})^2, \tag{1}$$

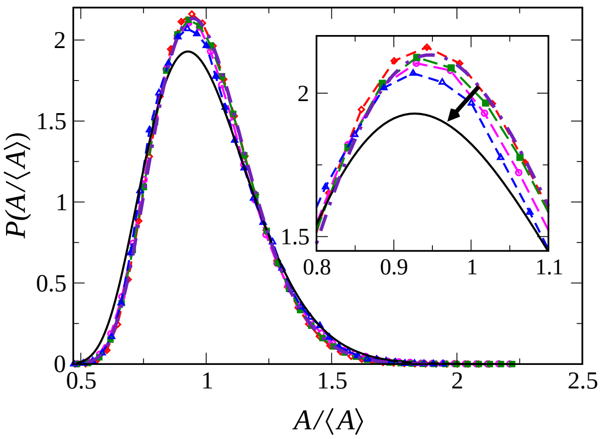

Figure S.1. (color online) Theoretical $P(A/\langle A\rangle)$ for reflected Bessel bridges and Bessel excursions for $d=4$ (black solid line) and $d=5$ (dot-dashed indigo line) along with simulations of $d=4$ SAW for $N=200$ (blue triangles), $N=480$ (dark green squares), $N=800$ (magenta circles), $N=1600$ (blue diamonds).

d	N range	$\nu_{calculated}$	$\nu_{predicted}$
2	$200-1600$	0.7507 ± 0.002	0.75 (exact)
3	$60-1002$	0.5890 ± 0.002	0.588
4	$200-2560$	0.5009 ± 0.002	0.5 (exact)

Table S.1. A summary of the scaling results for the exponent ν, comparing our simulations to known results. The value for $d=4$ was obtained via a logarithmic correction to scaling, as explained in the text.

where

$$\mathbf{r}_{c.m.} \equiv \frac{1}{N}\sum_{i=0}^{N-1}\mathbf{r}_i \tag{2}$$

and $A=\sum_{i=0}^{N-1}|\mathbf{r}_i-\mathbf{r}_0|$. For large N, $\bar{R}\equiv\sqrt{\langle R_g^2\rangle}$ increases as N^{ν_p} where ν_p is a critical exponent and as discussed in the text, $\nu_A=\nu_p+1$. However, we are mainly interested in the fluctuations of A, i.e., in the shape of the scaled distribution of $A/\langle A\rangle$ for different N. To check the reliability of the simulations we provide in Table S.1 the results obtained for $\bar{R}$. The $\nu_{calculated}$ results for $d=2$ and $d=3$ are equal within the error bars to those of $\nu_{predicted}$ while for $d=4$ $\nu_{calculated}$ is too large due to a logarithmic correction to scaling, which would become insignificant only for much larger N. In fact, our result for ν_p ($d=4$) (Table S.1) is equal within the error bars to the value of ν_p obtained by simulations of linear SAWs of up to $N=2400$;[3] there, a logarithmic correction to scaling has been studied leading to the optimal value of the exponent of the logarithm of $q=0.3125$ with $\nu_p=0.5005\pm0.0004$. Analyzing our results with this same q has led to a similar result.

The $d=4$ SAW case is a critical one, since the critical exponent ν_p for lower dimensions significantly differs from the $\nu_p=1/2$ of the non-interacting models, and for $d\geq4$ the interactions become unimportant for $N\to\infty$. Hence, we expect $P(A/\langle A\rangle)$ of the $d=4$ SAW to coincide with that of the non-interacting model. However, for finite N the interaction still has an effect on the distribution's shape, and an even more pronounced one for ring polymers. For the values of N we used in our SAW simulations the curve had not yet converged as can be seen in Fig. (S.1). A downward trend of the curves towards that of the non-interacting case (i.e. towards convergence) can nevertheless be seen. A similar problem is not found for SAW in $d=2,3$ which are reported in the main text.

Supplementary B: Numeric values of λ_k and d_k

The theoretical PDFs for different dimensions, presented in Eq. (10) in the paper, may be plotted using MATHEMATICA®. In order to find the numerical coefficients λ_k (eigenvalue) and d_k (the normalization coefficient of the eigenfunction) values

Dimension	Value	$k=1$	$k=2$	$k=3$	$k=4$	$k=5$	$k=6$	$k=7$
1, 3	λ_k^+	2.3381	4.088	5.2056	6.7871	7.944	9.02265	10.040
	d_k^+	1	1	1	1	1	1	1
1	λ_k^-	1.0188	3.2482	4.8201	6.1633	7.3722	8.4885	9.5345
	d_k^-	0.99088	0.5550	0.4554	0.4241	0.3837	0.3663	0.3566
2	λ_k^+	1.738	3.671	5.170	6.475	7.658	8.755	9.787
	d_k^+	1.1391	0.9195	0.8386	0.7885	0.7597	0.7317	0.7109
4	λ_k^+	2.873	4.494	5.868	7.098	8.231	9.291	10.294
	d_k^+	0.7585	0.8807	0.9523	1.0047	1.0482	1.0820	1.1108
5	λ_k^+	3.362	4.885	6.208	7.406	8.516	9.558	10.547
	d_k^+	0.5187	0.6762	0.7838	0.8646	0.9405	1.0012	1.0531

Table S.2. The first 7 eigenvalues, λ_k and the corresponding numeric coefficients d_k required for plotting the theoretical PDFs. The eigenvalues λ_k^+ of the $+$ solutions for $d=1$ and $d=3$ are the negatives of the zeros of the Airy function: $\mathrm{Ai}(-\lambda_k^+)=0$. The eigenvalues λ_k^- of the $-$ solution are the negatives of the zeros of its derivative: $\mathrm{Ai}'(-\lambda_k^-)=0$. The $d_k^\pm$ for $d=1$ are tabulated in Ref.[5]

of the kth mode, we used the numerical method described in detail in.[4] In Table S.2 we present the values of the first few λ_k and d_k for different boundaries in different dimensions. We found that the first 7 eigenvalues are usually sufficient for the evaluation of $P^{\pm}(A/\langle A\rangle)$.

References

1. Meirovitch, H. Statistical properties of the scanning simulation method for polymer chains. *J. Chem. Phys.* **89**, 2514–2522 (1988).
2. Baiesi, M. & Orlandini, E. Universal properties of knotted polymer rings. *Phyhs. Rev. E* **86**, 031805 (2012).
3. Rapaport, D. C. Self-avoiding walks in four dimensions: Logarithmic corrections to scaling. *Phys. Rev. B* **30**, 2906–2908 (1984).
4. Barkai, E., Aghion, E. & Kessler, D. A. From the area under the bessel excursion to anomalous diffusion of cold atoms. *Phys. Rev. X* **4**, 021036 (2014).
5. Abramowitz, M. & Stegun, I. A. *Handbook of Mathematical Functions* (National Bureau of Standards, Washington, 1964).
